# The Performance of Immunocytochemistry Staining as Triaging Tests for High-Risk HPV-Positive Women: A 24-Month Prospective Study

**DOI:** 10.1155/2020/6878761

**Published:** 2020-05-26

**Authors:** Yu-Cong Li, Yu-Qian Zhao, Ting-Yuan Li, Wen Chen, Guang-Dong Liao, Hai-Rui Wang, Hai-Ke Lei, Yue Guo, Qi Zhou

**Affiliations:** ^1^Department of Gynecology Oncology, Chongqing University Cancer Hospital, Chongqing 400030, China; ^2^Research Center of Cancer Prevention, Sichuan Cancer Hospital & Institute, Sichuan Cancer Centre, School of Medicine, University of Electronic Science & Technology of China, Chengdu 610041, China; ^3^Department of Epidemiology, National Cancer Center/National Clinical Research Center for Cancer/Cancer Hospital, Chinese Academy of Medical Sciences and Peking Union Medical College, Beijing 100021, China; ^4^Department of Gynecology and Obstetrics, The West China Second University Hospital, Sichuan University, Chengdu 610041, China; ^5^Shenzhen Center for Disease Control and Prevention, Shenzhen 518055, China; ^6^Department of Epidemiology, Chongqing University Cancer Hospital, Chongqing 400030, China; ^7^Department of Biomedical Informatics, University of Washington School of Medicine, Seattle 98195, USA

## Abstract

It is urgent to develop an accurate approach to improve the predictive performance of hrHPV-based screening. The aim is to evaluate the performance of p16/Ki-67 and p16/MCM2 staining to triage high-risk human papillomavirus- (hrHPV-) positive women. Cervical specimens were collected from eligible women and tested for hrHPV genotyping, cytology, p16/Ki-67, and p16/MCM2 staining at baseline. Women were invited to participate in follow-up screening by cytology and hrHPV testing at 24 months. Positive women received colposcopy and biopsies. Histopathological diagnoses were the gold standard. 485 women came back for the follow-up screening. The positive rate of p16/Ki-67 was 20.2% and of p16/MCM2 was 27.2%. The positive rates of p16/Ki-67 ( *P* < 0.001) and p16/MCM2 (*P*=0.021) were increased by the severity of histopathology findings. Among hrHPV-positive women, the sensitivity, specificity, PPV, and NPV for p16/Ki-67 were 90.9%, 67.0%, 16.5%, and 99.0%, and for p16/MCM2 were 81.8%, 43.1%, 9.4%, and 97.1%. The sensitivity of cytology for triaging hrHPV-positive women were lower than p16/Ki-67 (*P*=0.012) and p16/MCM2 (*P*=0.065). The cocktail staining did not add sensitivity to p16/Ki-67 or p16/MCM2 staining alone (*P* > 0.05), however, cutting down the specificity of p16/Ki-67 staining alone with statistical significance (67.0% vs. 40.2%, *P* < 0.001). The risk of CIN2+ within 24 months for hrHPV-positive but triaging negative women at baseline was 0.5 (0.1–2.7), 0.7 (0.1–4.1), and 2.4 (1.1–5.0) for p16/Ki-67, p16/MCM2, and cytology, respectively. As an objective and accurate immunocytochemical staining, the p16/Ki-67 and p16/MCM2 dual staining performed better than cytology to triage positive hrHPV. On condition that high-quality cytology is unavailable, immunocytochemical staining by p16/Ki-67 or p16/MCM2 is an option for triaging hrHPV-positive women. The combination of p16/Ki-67 and p16/MCM2 could not improve the accuracy in detecting CIN2+.

## 1. Introduction

Cervical cancer is one of the most common gynecological cancers worldwide. It caused 570,000 new cases and 311,000 deaths worldwide in 2018, of which 90% occurred in developing countries [1]. The mortality of cervical cancer has been reduced since the introduction of Pap smear. However, China bears a heavy disease burden from cervical cancer, especially in rural areas without adequate health resources [2]. Due to the high cost of the HPV vaccines, affordable and accurate screening remains the current option for population-based cervical cancer prevention within decades for most of the Chinese women.

High-risk human papillomavirus (hrHPV) detection has been included in the national cervical cancer screening system as a primary screening approach in North American and European countries [3]. However, it is not feasible to refer all hrHPV-positive women to colposcopy. Most HPV infections are transient and a single HPV DNA test cannot distinguish transforming infections from transient ones. Cytology has been recommended as a triaging test for HPV-based screening [4]. However, due to the lack of experienced cytologists in resource-limited regions, it is difficult to build a hrHPV-based screening system with high-quality cytology triaging. It is urgent to develop an accurate approach to improve the predictive performance of primary screening and reduce the number of unnecessary colposcopies.

Studies have demonstrated that p16^INK4a^ (p16) and Ki-67 are optional biomarkers of dysplasia in cervical cytology preparations [5–7]. The p16/Ki-67 dual staining was designed to detect the coexpression of p16 and Ki-67 in cells. A concurrent cytological p16 and Ki-67 staining could be an efficient tool to triage women with atypical squamous cells of undetermined significance (ASC-US) or low-grade squamous intraepithelial lesion (LSIL) cytology [8]. Minichromosomal maintenance protein 2 (MCM2) participates in DNA replication in all eukaryotic cells. It promotes cell proliferation by loading the complex onto DNA and unwinding the DNA helicase to permit DNA synthesis.

The performance of immunocytochemical staining assays has been reported in cross-sectional studies as an accurate triaging tool [8, 9]. However, prospective data are necessary to evaluate the protection among the Chinese population. This study was conducted to evaluate the performance of a cocktail immunocytochemical staining by p16/Ki-67 and/or p16/MCM2 for detecting high-grade cervical intraepithelial neoplasia (CIN) among hrHPV-positive women.

## 2. Materials and Methods

### 2.1. Population and Procedures

From April 2015 to May 2016, 4,070 eligible women in Wanzhou District, Chongqing, and Shuangliu County, Chengdu, China, were recruited in the national cervical cancer program. The inclusion criteria were aged 35–64 years, no history of cervical diseases, had an intact cervix, not pregnant currently, understood the study procedure, and were able to provide written informed consent. The study was approved by the Institutional Review Boards of the Chongqing University Cancer Hospital (no. 2014010) and West China Second University Hospital of Sichuan University (no. K2014018).

The cervical exfoliated cells were obtained with a cytobrush by physicians and stored in ThinPrep PreservCyt Solution (Hologic Inc. San Diego, U. S.) for HPV genotyping and cytology. At baseline, all women have had HPV genotyping. Women tested positive for HPV16/18 or other 13 hrHPV subtypes (31, 33, 35, 39, 45, 51, 52, 56, 58, 59, 66, 68, and 82) positive with reflex cytology ASC-US or worse were referred to colposcopy. P16/Ki-67 (MXB, Fuzhou, China) and p16/MCM2 (MXB, Fuzhou, China) immunocytochemical dual staining was performed for hrHPV-positive women and a 12% random selection of hrHPV negative women at baseline, whereas the management of the women was not based on the immunocytochemical stain results.

All enrolled women were invited to participate in the 24-month follow-up screening, except for those diagnosed as CIN2+ at baseline and were screened by hrHPV genotyping and cytology cotesting. Women with positive hrHPV genotyping or ASC-US + cytology at follow-up screening would be referred to colposcopy.

### 2.2. HPV DNA Testing

A 1 ml aliquot removed from ThinPrep PreservCyt Solution (Hologic Inc., Bedford, U. S.) was detected by HPV Genotyping Real-Time PCR Kit (Liferiver, Shanghai, China). The Liferiver PCR Kit detects HPV DNA by nucleic acid hybridization with a pooled probe set of 15 hrHPV types, including HPV16, HPV18, and other 13 high-risk subtypes. The kit shows equal analytical and clinical accuracy compared with Cobas 4800 [10]. All reactions were performed in a 40 *μ*l volume using the ABI PRISM 7000 (Applied Biosystems, US). Each reaction contained a 36 *μ*l mixture of 2 × TaqMan universal PCR master mix with uracil-N-glycosylase (Applied Bio-Systems, US) and two fluorescent probes. The amplification profile was initiated by a 2-minute incubation at 94°C, followed by a two-step amplification of 10 seconds at 93°C and 31 seconds at 62°C for 40 cycles.

### 2.3. Liquid-Based Cytology

The sample preparation process was performed according to the instructions of the manufacturer. Cells were fixed with 95% ethanol and stained using the Papanicolaou method. The slides were interpreted by two experienced cytotechnologists and confirmed by a third pathologist according to the classification of the 2001 Bethesda nomenclature, including negative for intraepithelial lesion or malignancy (NILM), ASC-US, LSIL, the high-grade squamous intraepithelial lesion (HSIL), atypical squamous cells, favor high grade (ASC-H), atypical glandular cells (AGC), and squamous cell carcinoma (SCC). Cytology abnormality as ASC-US or worse (ASC-US+) was deemed as positive, which led to a referral to colposcopy and biopsy procedure.

### 2.4. Immunocytochemistry Staining

Two slides were prepared from the PreservCyt specimen for p16/Ki-67 and p16/MCM2 staining, respectively. The kit of p16/Ki-67 staining contains a cocktail antibody, comprising of mouse monoclonal antibody (clone MX007) targeting on p16 protein and rabbit monoclonal antibody (clone MIB-1) targeting on Ki-67 protein. The cytology slide was counterstained by hematoxylin by a 3-step procedure including 85%, 95%, and pure ethanol medium, each for 1 minute. The kit of p16/MCM2 staining was composed of p16 antibody (clone MX007) and rabbit anti-human MCM2 monoclonal antibody (clone SP85). Slides with at least one cervical epithelial cell that dual positive for red cytoplasmic immunostaining (p16) and brownish nuclear immunostaining (Ki-67) or moderate to intense yellow-brown nuclear staining (MCM2) were defined as positive, while those without any double-stained cells were deemed as negative. All slides were viewed by a trained cytotechnologist blinded to other results.

### 2.5. Colposcopy and Histopathology

Colposcopy was performed in local clinics within one month. A colposcopy-directed biopsy was performed under a positive colposcopic finding. Endocervical curettage was performed (ECC) if necessary. The histopathology findings were reported according to the CIN systems. All the CIN and a 10% random sample of negative slides were reviewed by a panel of senior pathologists from Chongqing Cancer Hospital and the Second Affiliated Hospital of Sichuan University. Women with CIN2, CIN3, squamous cell carcinoma (SCC), adenocarcinoma in situ (AIS), and adenocarcinoma (ADC) results were defined as CIN2+ cases. The final diagnosis was based on the consensus of the panel review.

### 2.6. Statistical Analysis

The positive rate for the immunocytochemistry staining was stratified by hrHPV genotyping (negative, HPV 16/18+, or other subtypes positive), cytology (NILM, ASC-US, LSIL, and HSIL+: ASC-H, HSIL), and histopathology (normal/CIN1, CIN2, and CIN3+) to test the trend. Sensitivity, specificity, positive predictive value (PPV), and negative predictive value (NPV), area under the receiver operating characteristic curve (AUC) for detecting CIN2+ with 95% confidence interval (CI) were calculated to compare the diagnostic performance of cytology and dual staining as triaging hrHPV-positive women. Risk and relative risk (RR) with 95%CI were evaluated. All data analyses were performed using SPSS 23.0. All statistical tests were two-sided, and *P* values less than 0.05 were considered statistically significant.

## 3. Results

**Figure 1:**
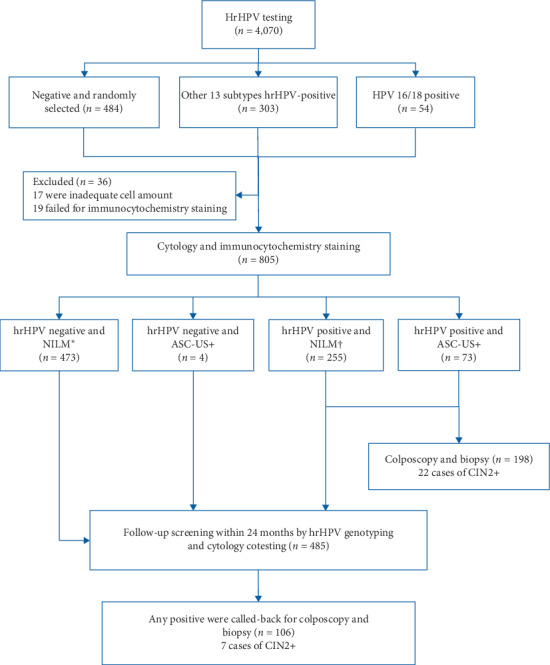
Flow diagram showing procedure and test results. ^*∗*^Including 16 with unsatisfactory cytology. ^†^11 with unsatisfactory cytology.

The flowchart of the study is shown in Figure 1. At baseline, 357 (8.8%) women from the 4,070 screened women with positive hrHPV and another 484 hrHPV-negative were randomly selected for immunocytochemistry staining. Finally, 805 women were included for data analysis after excluding 36 women who failed in immunocytochemistry staining. The clinical results for baseline and the 24-month follow-up screening are shown in Table 1. Among the 805 women, 40.7% (328/805) were hrHPV-positive. Among hrHPV-positive women, 12.2% (40/328) were HPV 16/18 positive, 277 84.5% (277/328) were other 13 subtypes of hrHPV-positive, and 11 (3.4%) were multiple infected by HPV 16/18 and other hrHPV subtypes. Twenty-seven women have had unsatisfactory cytology at baseline. Seventy-seven (9.6%) women were deemed as ASC-US or worse, including 45 ASC-US (58.4%), 24 LSIL (31.2%), 4 ASC-H (5.2%), and 4 HSIL (5.2%). 163 (20.2%) women were p16/Ki-67 positive and 223 (27.7%) were p16/MCM2 positive, respectively. Among the 328 hrHPV-positive women, 198 (60.4%) women finished the colposcopy examination and the CIN2+ yield of the population was 2.7% (22/805) at baseline. For hrHPV negative women at baseline, 95.8% (457/477) were cytology NILM, and 87.4% (417/477) and 89.5% (427/477) of the hrHPV negative women have had negative p16/Ki-67 or p16/MCM2 staining, respectively.

At the 24 months, 61.9% (485/783) women with negative or CIN1 findings at baseline came back for the follow-up screening. Among them, 4.7% (23/485) were hrHPV-positive with cytology ASC-US+, 3.3% (16/485) were cytology ASC-US+ with negative hrHPV, and 17.5% (85/485) were hrHPV with negative cytology. In total, 106 women have had colposcopy and biopsy. Seven new cases of CIN2+ were detected; the yield was 1.4% (7/485). No statistical significance between the women followed-up and loss to follow-up were found for the baseline primary screening results (hrHPV: 37.5% vs. 41.6%, *P*=0.26; cytology: 8.5% vs. 8.4%, *P*=0.98; p16/Ki-67 : 19.2% vs. 16.8%, *P*=0.40; p16/MCM2: 26.6% vs. 25.5%, *P*=0.61). Six out of the 7 CIN2+ cases were other hrHPV subtypes positive with NILM cytology. Among the 6 cases, 3 women attended the referral examination but were negative under colposcopy or normal/CIN1 biopsy, other 3 women did not attend the referral colposcopy at baseline. The other 1 case of CIN2+ was p16/MCM2 staining positive alone at baseline.

As shown in Table 2, for HPV 16/18 positive women including the 11 multi-infected women, 49.0% (25/51) were p16/Ki-67 positive and 68.6% (35/51) were p16/MCM2 positive, which were statistically higher than hrHPV negative women (49.0% vs. 8.8%, *P* < 0.0001; 68.6% vs. 6.5%, *P* < 0.01). For women with other hrHPV subtypes positive, 34.7% (96/277) were p16/Ki-67 positive and 56.7% (157/277) were p16/MCM2 positive, which were statistically higher than hrHPV negative women as well (34.7% vs. 8.8%, *P* < 0.01; 56.7% vs. 6.5%, *P* < 0.0001). The positive rate for p16/Ki-67 and p16/MCM2 among HPV 16/18 and other subtypes of hrHPV showed no significance although with a borderline *P* value for p16/Ki-67 (49.0% vs. 34.7%, *P*=0.051; 68.6% vs. 56.7%, *P*=0.11). 90.9% (20/22) of the CIN2+ were p16/Ki-67 positive, 81.8% (18/22) were p16/MCM2 positive. The positive rates of p16/Ki-67 and p16/MCM2 staining were increased by the severity of cytology (*P* < 0.001) and histopathology findings (*P* < 0.001, *P*=0.021).

Sensitivity, specificity, PPV, NPV, and AUC of cytology and immunocytochemistry staining for detecting CIN 2+ among hrHPV-positive women are shown in Table 3. The sensitivity, specificity, PPV, and NPV for cytology were 50.0%, 79.7%, 15.1%, and 95.7%, for p16/Ki67 were 90.9%, 67.0%, 16.5%, and 99.0%, and for p16/MCM2 were 81.8%, 43.1%, 9.4%, and 97.1%. The sensitivity of cytology for triaging hrHPV-positive women was statistically lower than p16/Ki-67 (*P*=0.012) and not significantly lower than p16/MCM2 (*P*=0.065). However, the specificity of p16/Ki67 and p16/MCM2 was significantly lower than cytology (*P* < 0.001). The PPV of cytology and p16/Ki67 staining were comparable (15.1% vs. 16.5%, *P*=0.012). The cocktail staining of p16/Ki-67 and p16/MCM2 did not add sensitivity to p16/Ki-67 staining, however, cutting down the specificity of p16/Ki-67 staining alone with statistical significance (67.0% vs. 40.2%, *P* < 0.001). Besides, the sensitivity and specificity of p16/MCM2 staining alone were similar with the cocktail staining (81.8% vs. 90.9%, *P*=0.38; 43.1% vs. 40.2%, *P*=0.46).

Table 4 shows the 24-month risk and RR of CIN2+ for one-time baseline screening among the follow-up population. The risk of developing CIN2+ lesion within 24 months for negative hrHPV, p16/Ki-67, and p16/MCM2 women at baseline were 0.3 (95% CI: 0.1–1.9), 0.5 (95% CI: 0.1–1.8), and 0.3 (95% CI: 0.1–1.6), respectively. However, it for women with NILM cytology at baseline was 1.6 (95% CI: 0.8–3.2), which was much higher than the dual staining. For positive primary screening but negative of CIN2+ at baseline, the risk was 5.4 (95% CI: 2.3–12.0), 4.7 (95% CI: 2.2–9.8), and 3.3 (95% CI: 1.5–7.0) for p16/Ki-67 positive, p16/MCM2 positive, and hrHPV-positive, respectively. RR for hrHPV was 10.0 (95% CI: 1.2–82.3), for p16/Ki-67 was 10.5 (95% CI: 2.1–53.5), for p16/MCM2 was 16.6 (95% CI: 2.0–136.2), and for p16/Ki-67 and p16/MCM2 was 12.8 (95% CI: 1.6–105.2), respectively.

## 4. Discussion

The screening strategy of hrHPV testing cotested with or triaged by cytology has been recommended for cervical cancer screening in many countries [11, 12]. HR-HPV testing has high sensitivity and can detect over 90% CIN2+ cases reported by previous studies [13–15]. However, since most HPV infection is transient and only persistent infection leads to cervical cancer, the specificity of hrHPV testing in detecting high-grade cervical lesions and cancer is lower than cytology. To maximize benefits and minimize potential harm are the principles of a screening strategy. Since the unsatisfactory specificity for hrHPV testing as primary screening and the difficulty to build a qualified cytology screening system, developing objective testing for triaging is required. The presented data imply that the p16/Ki-67 and p16/MCM2 staining are promising to triage hrHPV-positive women with high sensitivity and low risk for negative women [16].

P16^INK4a^ is a cyclin-dependent kinase inhibitor that has been proven to be significantly overexpressed in transforming infections with hrHPV. As a negative regulator of cell proliferation, p16 protein can downregulate the activity of CDK4 and CDK6 once the retinoblastoma protein has been inactivated. It has been recognized to be a surrogate marker for cervical precancerous lesions [17]. However, the overexpression of p16 may also be observed in tubal metaplasia and atrophic cells as well as in normal columnar cells from the cervix, which leads to unsatisfactory specificity [18]. Ki-67 is a nuclear antigen that can be detected in the non-G0 phase of the cell cycle, indicating the process of cell proliferation [19]. The coexpression of p16 and Ki-67 within the same cell indicates the transformation of cervical epithelial cells, which could progress into cancer. Minichromosomal maintenance protein 2 participates in DNA replication in all eukaryotic cells. It promotes cell proliferation by loading the complex onto DNA and unwinding the DNA helicase to permit DNA synthesis [20, 21].

Cross-sectional studies have reported the accuracy of p16/Ki-67 and p16/MCM2 dual staining as a primary or triaging test for detecting high-grade cervical lesions including the Chinese population [22–24]. Zhang et al. find that the positive rate of p16/Ki-67 staining increased significantly with histological severity. The sensitivity of p16/Ki-67 in detecting CIN2+ as the primary screening was 88.10%. The specificity was 85.02% for detecting CIN2+, which was similar to cytology (84.71% for CIN2+) but higher than hrHPV as primary screening [22]. Another study reported a sensitivity of p16/Ki-67 dual-stain testing for CIN2+ in triaging Pap negative/HPV positive women was 91.9%, and specificity was 82.1% for CIN2+ [23]. Wentzensen et al. reported p16/Ki-67 was positive in 82.8% of CIN2 and 92.8% in CIN3. The sensitivity and specificity to detect CIN3+ were 97.2% and 60.0% among women 30 years and older [24]. In our data, the performance of cytology to triage hrHPV-positive women was unsatisfactory, of which the sensitivity was 50.0% (95% CI: 30.7%–71.2%), and the NPV was 95.7% (95% CI: 92.4–97.6). The sensitivity of p16/Ki-67 and p16/MCM2 was much higher than cytology as 90.9% (95% CI: 72.2–97.5) and 81.8% (95% CI: 61.5–92.7), with NPV 99.0% (95% CI: 96.6–99.7), and 97.1% (95% CI: 92.7–98.9), respectively. In the case of no high-quality cytology was available, the objective dual staining of p16/Ki-67 or p16/MCM2 could be a substitute test.

The strength of our study is the prospective study design that makes it possible to evaluate the risk of CIN2+ lesions for negative women at baseline. The diagnostic bias was attempted to minimize by cotesting of hrHPV testing and cytology at follow-up screening. Any positive women were referred for colposcopy and/or biopsy procedure to detect any CIN2+ cases at the follow-up screening. The limitations of the study include the unsatisfactory follow-up rate at the 24-month screening, in which only 61.9% of women came back for the cotesting. However, the baseline positive rates of hrHPV, cytology, p16/Ki-67, and p16/MCM2 were similar between the women lost to follow-up and followed-up, which may not affect the evaluation of the risk and RR. Another limitation is the relatively short time interval for follow-up screening. Since the 6 new CIN2+ cases were associated with other hrHPV subtypes infection and NILM cytology at baseline, it may take longer than 24 months for them to develop a CIN2+ [25, 26], which we attribute the 6 new CIN2+ more likely to be missed cases at baseline by false-negative colposcopy or loss to the called-back colposcopy, rather than newly developed CIN2+ cases within 24 months. However, the data of risk were also of clinical significance for the management of screened women.

## 5. Conclusions

In conclusion, p16/Ki-67 and p16/MCM2 dual staining shows good clinical performance in detecting CIN2+ with higher sensitivity and protection than cytology and could be considered as an efficient triage method to manage women with positive hrHPV. The combination of p16/Ki-67 and p16/MCM2 could not improve the accuracy in detecting CIN2+.

## Figures and Tables

**Table 1:** Clinical results of the enrolled women at baseline and at follow-up.

Histopathology		hrHPV			Cytology^*∗*^		p16/Ki-67		p16/MCM2	
		Negative, (*n*/*N* %)	16/18 positive, (*n*/*N*, %)	Other subtypes positive, (*n*/*N*, %)	NILM (*n*/*N*, %)	ASC-us+ (*n*/*N*, %)	Negative, (*n*/*N*, %)	Positive, (*n*/*N*, %)	Negative, (*n*/*N*, %)	Positive, (*n*/*N*, %)
Baseline	Not biopsied or normal (*N* = 758)	477 (62.9)	36 (4.7)	245 (32.3)	672 (88.7)	59 (7.8)	626 (82.6)	131 (17.3)	570 (75.2)	188 (24.8)
	CIN1 (*N* = 25)	0 (0.0)	8 (32.0)	17 (68.0)	18 (72.0)	7 (28.0)	13 (52.0)	12 (48.0)	8 (32.0)	17 (68.0)
	CIN2+ (*N* = 22)	0 (0.0)	7 (31.8)	15 (68.2)	11 (50.0)	11 (50.0)	2 (9.1)	20 (90.9)	4 (18.2)	18 (81.8)
	Total (*N* = 805)	477 (59.3)	51 (6.3)	277 (34.4)	701 (87.1)	77 (9.6)	642 (79.8)	163 (20.2)	582 (72.3)	223 (27.7)

Follow-up	Not biopsied or normal (*N* = 472)	377 (79.7)	11 (2.3)	84 (17.8)	396 (83.9)	33 (7.0)	—	—	—	—
	CIN1 (*N* = 6)	0 (0.0)	1 (16.7)	5 (83.3)	1 (16.7)	4 (66.7)	—	—	—	—
	CIN2+ (*N* = 7)	0 (0.0)	1 (14.3)	6 (85.7)	5 (71.4)	2 (28.6)	—	—	—	—
	Total (*N* = 485)	377 (77.7)	13 (2.7)	95 (19.6)	402 (82.9)	39 (8.0)	—	—	—	—

^*∗*^27 women at baseline and 44 at follow-up screening with unsatisfactory cytology are not shown.

**Table 2:** Positive rate of p16/Ki-67, p16/MCM2 and cocktail staining by hrHPV genotyping, cytology, and histopathology at baseline.

		p16/Ki-67		p16/MCM2		Cocktail^†^	
		Negative, *n* (*n*/*N* %)	Positive, *n* (*n*/*N* %)	Negative, *n* (*n*/*N* %)	Positive, *n* (*n*/*N* %)	Negative, *n* (*n*/*N* %)	Positive, *n* (*n*/*N* %)
HrHPV	Negative (*N* = 477)	435 (91.2)	42 (8.8)	446 (93.5)	31 (6.5)	415 (87.0)	62 (13.0)
	HPV 16/18+ (*N* = 51)	26 (51.0)	25 (49.0)	16 (31.4)	35 (68.6)	14 (27.5)	37 (72.5)
	Other subtypes+ (*N* = 277)	181 (65.3)	96 (34.7)	120 (43.3)	157 (56.7)	111 (40.1)	166 (59.9)

Cytology^*∗*^	NILM (*N* = 701)	588 (83.9)	113 (16.1)	531 (75.7)	170 (24.3)	497 (70.9)	204 (29.1)
	ASC-US (*N* = 45)	18 (40.0)	27 (60.0)	14 (31.1)	31 (68.9)	13 (28.9)	32 (71.1)
	LSIL (*N* = 24)	10 (41.7)	14 (58.3)	10 (41.7)	14 (58.3)	7 (29.2)	17 (70.8)
	HSIL+ (*N* = 8)	3 (37.5)	5 (62.5)	2 (25.0)	6 (75.0)	1 (12.5)	7 (87.5)

Histopathology	Normal/CIN1 (*N* = 783)	640 (81.7)	143 (18.3)	578 (73.8)	205 (26.2)	538 (68.7)	245 (31.3)
	CIN2 (*N* = 7)	1 (14.3)	6 (85.7)	2 (28.6)	5 (71.4)	1 (14.3)	6 (85.7)
	CIN3+ (*N* = 15)	1 (6.7)	14 (93.3)	2 (13.3)	13 (86.7)	1 (6.7)	14 (93.3)

^†^Cocktail staining positive means that either the p16/Ki67 or p16/MCM2 positive. ^*∗*^Cytology HSIL + including HSIL and ASC-H.

**Table 3:** Performance of p16/Ki-67, p16/MCM2 staining, and cytology for triaging hrHPV-positive women in detecting CIN2+ at baseline.

Triage tests	Sensitivity, % (95% CI)	Specificity, % (95% CI)	PPV, % (95% CI)	NPV, % (95% CI)	AUC (95% CI)
HrHPV-positive women at baseline (*n* = 328)					
Cytology	50.0 (30.7–71.2)	79.7 (74.9–83.9)	15.1 (8.6–25.0)	95.7 (92.4–97.6)	0.65 (0.52, 0.78)
p16/Ki-67	90.9 (72.2–97.5)	67.0 (61.5–72.0)	16.5 (11.0–24.2)	99.0 (96.6–99.7)	0.79 (0.71, 0.87)
p16/MCM2	81.8 (61.5–92.7)	43.1 (37.7–48.7)	9.4 (6.0–14.3)	97.1 (92.7–98.9)	0.63 (0.52, 0.74)
Cocktail staining^*∗*^	90.9 (72.2–97.5)	40.2 (34.9–45.7)	9.9 (6.5–14.7)	98.4 (94.4–99.6)	0.66 (0.56, 0.76)

^*∗*^Cocktail staining positive means that either the p16/Ki-67 or p16/MCM2 positive.

**Table 4:** Risk and relative risk of CIN2+ for baseline hrHPV, cytology, p16/Ki-67, and p16/MCM2 staining within 24-months.

Screening tests at baseline		CIN2+		24 months risk for new CIN2+, % (95% CI)	RR_N_ (95% CI)
		Cumulative cases within 24 months, *N*_C_	New cases at follow-up screening, *N*_N_		
HrHPV	Positive	28	6	3.3 (1.5–7.0)	10.0 (1.2–82.3)
	Negative	1	1	0.3 (0.1–1.9)	

Cytology	ASC-US+	11	0	0.0 (0.0–8.6)	—
	NILM	18	7	1.6 (0.8–3.2)	

p16/Ki-67	Positive	25	5	5.4 (2.3–12.0)	10.5 (2.1–53.5)
	Negative	4	2	0.5 (0.1–1.8)	

p16/MCM2	Positive	24	6	4.7 (2.2–9.8)	16.6 (2.0–136.2)
	Negative	5	1	0.3 (0.1–1.6)	

Cocktail staining^*∗*^	Positive	26	6	3.9 (1.8–8.2)	12.8 (1.6–105.2)
	Negative	3	1	0.3 (0.05–1.7)	

hrHPV+ & cytology	Positive	11	0	0.0 (0–5.0)	—
	Negative	17	6	2.4 (1.1–5.0)	

hrHPV+& p16/Ki-67	Positive	25	5	4.1 (1.8–9.3)	8.6 (1.01–72.4)
	Negative	3	1	0.5 (0.1–2.7)	

hrHPV+ & p16/MCM2	Positive	23	5	2.6 (1.1–6.0)	3.5 (0.4–30.0)
	Negative	5	1	0.7 (0.1–4.1)	

hrHPV+ & cocktail^*∗*^	Positive	25	5	2.5 (1.1–5.6)	3.1 (0.4–26.0)
	Negative	3	1	0.8 (0.1–4.4)	

^*∗*^Cocktail staining positive means that either the p16/Ki67 or p16/MCM2 positive.

## Data Availability

The datasets used and/or analyzed during the present study are available from the first author and corresponding author upon request.
